# Characteristics and Trends in Hypnotics Consumption in the Largest Health Care System in Israel

**DOI:** 10.1155/2016/8032528

**Published:** 2016-08-31

**Authors:** O. Marom, G. Rennert, N. Stein, K. Landsman, G. Pillar

**Affiliations:** ^1^Sleep Clinic, Clalit Health Services, Technion Faculty of Medicine, Haifa, Israel; ^2^Department of Community Medicine and Epidemiology, Carmel Medical Center and Technion Faculty of Medicine, Haifa, Israel; ^3^Office of Chief Physician, Clalit Health Services, Tel Aviv, Israel

## Abstract

*Objectives*. To quantify and characterize hypnotics consumption habits among adult patients insured by Clalit Health Services (CHS), the largest health care provider in Israel, in 2000 and 2010.* Methods*. A retrospective analysis of CHS computerized pharmacy records. Data were collected for all patients over the age of 18 years who were prescribed hypnotics in 2000 and in 2010.* Results*. Sleep medications were consumed by 8.7% of the adult CHS population in 2000 and by 9.6% in 2010. About one-quarter of consumers were treated for more than 6 months in both years. Multiple sleeping drugs were consumed more often in 2010 (45.2%) than a decade before (22%). While in 2000 benzodiazepines accounted for 84.5% of hypnotics, in 2010 this was reduced to 73.7% (*p* < 0.05). Of all patients treated for longer than 6 months only 11% in 2000 and 9% in 2010 required a dose escalation suggesting the absence of tolerance.* Conclusions*. Nine percent of the Israeli population consumes hypnotics. There is a major increase in prescription of combination of medications between 2000 and 2010, with an increase in Z class medications use and reduction in benzodiazepines. Most patients chronically treated did not escalate dosage, suggesting the absence of tolerance.

## 1. Introduction

Insomnia is the most common sleep disorder. The prevalence of insomnia in various studies was reported to be 10–30% in the general adult population in various regions of the world [1–6]. Major factors that are associated with higher rates of Insomnia include female gender, older age, mental illness, insomnia in the past, sedentary lifestyle, and comorbid illness [2, 7].

It is well established that insomnia is associated with morbidity and a decrease in daily function and quality of life. Several diseases were reported to be strongly associated with insomnia. These include ischemic heart disease, hypertension, chronic pain, diabetes, respiratory, genitourinary, and gastrointestinal abnormalities, decreased immunity, and mental and cognitive problems [2, 8–13]. Insomnia is also associated with reduced productivity at work and increased health care utilization [2, 7, 14]. The direct and indirect cost of Insomnia was estimated to be 65 billion dollars per year [8]. Treating insomnia is thus highly important, has a positive effect on individuals, and is cost effective. For example, treating insomnia in patients with diabetes without changing anything else has led to improvement in diabetic control [15].

Treatment options include sleep hygiene issues, cognitive behavioral treatment, other relaxation techniques, medications, or a combination of these. The most commonly used prescription medications include benzodiazepines, sedative antidepressants, and Z class drugs. The latter are considered more selective for the alpha 1 subunit in the GABA receptor and therefore potentially cause less side effects, addiction, and tolerance [1, 3, 4]. Indeed, based on clinical observations and some studies, addiction and tolerance with the recommended doses of Z class medications are rare [7, 16, 17]. Yet, some reports have linked the usage of hypnotics with increased morbidity and mortality [18–20].

Only a few reports have addressed the issue of hypnotics usage in the general population. Recently, in a cross-sectional study surveying 32,328 noninstitutionalized community-dwelling U.S. adults in the USA, it has been reported that only about 3% of the population were using hypnotics (eszopiclone, zaleplon, zolpidem, estazolam, flurazepam, quazepam, temazepam, triazolam, amobarbital, amobarbitalsecobarbital, chloral hydrate, doxepin, quetiapine, ramelteon, and trazodone). Zolpidem and trazodone were used most commonly. They reported increase of usage over the years, with increase in combination treatments [21]. Hypnotic usage was more common among older people and among those with mental illness. The Canadian national health survey has reported that between 1994 and 2003 hypnotics usage has doubled, reaching 5.5% of the population in 2003 [20]. Interestingly they reported a large tendency for hypnotic usage among morbidly obese men.

Clalit health services (CHS) is the largest health insurance provider in Israel, insuring more than 4 million people. It is important to emphasize that in Israel there is a national health insurance policy and all the Israeli population is insured by 4 major providers. The decision where medications are funded by health insurance is made nationally and is not affected by the type of health insurance provider. CHS uses Patient Electronic Medical Record for more than 25 years. The database is very well kept and several previous studies have utilized partial data of this registry in the field of sleep, predominantly sleep apnea [22–26]. There is a very prompt data-base management in this closed population, providing accurate and representative information. The system is nationally managed, and any medication consumption whether it was prescribed by the primary care physician or by a specialist or in another city is recorded into the database. Thus, this database is highly representative and reliable. Since data regarding hypnotic usage habits are sparse in general and are lacking in Israel, we sought to utilize this huge database and to investigate it for both current hypnotic usage and trends over the years.

## 2. Methods

### 2.1. Study Population and Database

This is a retrospective analysis of data from the computerized pharmacy records of CHS. Clalit Health Care Services has a cumulative reliable and quality-controlled database. Physicians enter data during hospital or community setting clinic visits. This database is highly reliable and contains all physician visits, all prescribed medications, and over 98% of all patient diagnoses [22–27]. The Clalit Health Services national pharmacy database is a very well managed database consisting of a full annotation of every prescription dispensed by any of the roughly 4 million insured by CHS with details on class of medication, generic name, commercial name, and dosage. These data are combined with other databases of CHS including the demographic database and the health and illnesses status of all CHS insurees. Data were collected for all patients over the age of 18 years who were prescribed with hypnotics in 2000 and in 2010. The study was approved by the institutional review board of Clalit Health Services at Carmel Medical Center in Haifa.

The primary aim of the study was to identify and characterize the usage of hypnotics, in the years 2000 and 2010. The use of the following medications was sought: benzodiazepines: brotizolam, lorazepam, clonazepam, nitrazepam, and flunitrazepam; sedative antidepressants: mirtazapine, amitriptyline, trazodone, Z class drugs: zolpidem, and zopiclone (eszopiclone and zaleplone are not in use in Israel).

Data collected included:

1. Demographic details: age, gender, and sector (Arab, highly religious Jews, all others). There are two major minorities in Israel: the Arab population (about 20.3% of the general adult population) and highly Orthodox Jews (about 3% of the general adult population).
2. Data regarding hypnotics consumption: duration of consumption in months (1, 1–3, 6+), percentage of consumers of only one type of hypnotic, consumption distribution according to medication group type, and dosage changes.

The second aim was to test for tolerance, expressed as dose increment in patients using hypnotics for longer than 6 months. Since the CHS policy is of monthly prescription for these medications, we included people who dispensed between 6 and 13 prescriptions, for the same type of sleeping medication, with the same dosage in mg, with at least 6-month interval between first and last prescriptions during the years 2000 and 2010. Increase or decrease in the amount consumed was defined by change of 10 pills or more between first and last prescriptions.

### 2.2. Statistical Analyses and Ethical Issues

No names or personal identity details were included. Data were analyzed using SPSS software. The statistics consisted of predominantly descriptive statistics and also comparative analyses (*t*-test) between the various periods or groups. Chi-square analysis was utilized to compare proportions. *p* < 0.05 was considered statistically significant.

## 3. Results

### 3.1. Demography and Comparison of Sleep Medication Consumers for Years 2000 and 2010

In 2000, 222,274 people out of 2,561,990 adults insured at CHS consumed sleep medications (8.7%). In 2010, 282,975 people out of 2,936,947 adults insured at CHS consumed sleep medication (9.6%). The demographic characteristics of these people, as well as their hypnotics consumption habits, are presented in Tables 1 [Table tab2] [Table tab3] [Table tab4] [Table tab5]–6, comparing the years 2000 and 2010. The average and median age of hypnotics usage was significantly lower in 2010 than in 2000, by approximately 7 years (*p* < 0.0001, Table 1), despite the fact that the median and average age of CHS population (and the general population) steadily increased during this period. As for gender effects, in both years 2000 and 2010, 64% of hypnotics consumers were females, and only 36% were males (*p* < 0.0001 for gender effect, NS for time difference).

The percentage of hypnotics usage among Orthodox Jews was significantly lower (in both 2000 and 2010) than all others, and the percentage of hypnotics usage among Arabs was significantly lowest in both 2000 and 2010 (*p* < 0.0001 for all comparisons, Table 2).

In both 2000 and 2010 most people consumed sleep medications for short periods (less than 3 months), and only 21–25%, respectively, consumed sleep medication for longer than 6 months (Table 3). Average age was higher for people that consumed sleep medications for longer periods (Table 3).

In both 2000 and 2010 chronic use (6+ months) within the Arab sector was significantly lower than in other sectors (Table 4). The changes within ethnic groups from 2000 to 2010 indicate an increase in chronic usage in the Arab population (*p* < 0.005), but still dramatically lower than their proportion in the general population and in their proportion in CHS insured.

#### 3.1.1. Percentage of Consumers of Only One Type of Sleep Medication

In 2000 78% of sleep medication consumers were prescribed with solely a single medication. In 2010 there was a dramatic decrease to only 54.8% of sleep medication consumers that were prescribed with a single medication. In other words there was a substantial increase in prescription of combination of medications from 2000 to 2010 (from 22% to 45.2%).

In 2000 benzodiazepines and Z drugs accounted for 84.5% and 2.1% of hypnotics, respectively. In 2010 benzodiazepines were reduced to 73.7% (*p* < 0.0001) and Z drugs accounted for 9.8% (*p* < 0.001, Table 5). The percentage usage of antidepressants increased from 13.4% in 2000 to 16.5% in 2010 (*p* < 0.005, Table 5).

### 3.2. Change in the Quantity of Sleep Medications Consumption during Long-Term Usage (More Than 6 Months)

During years 2000 and 2010, 47,688 and 36,546 people, respectively, dispensed between 6 and 13 prescriptions, for the same type of sleep medication, with the same dosage in mg, with at least 6-month interval between the first and last prescriptions. Increase or decrease in the amount of medication consumed was a priori defined (clinically) by a change of 10 pills or more (change in dosage of 30%) between the first and the last prescriptions (6 or more months apart). Of all patients treated for longer than 6 months only 11% in 2000 and 9% in 2010 required a dose increase, suggesting most patients did not develop tolerance (Table 6). Of note, 7% and 6% of the patients had actually decreased the number of pills consumed over the long-term usage, indicating either basic improvement in their insomnia and/or reducing hypnotics of one type for a combination therapy with another type. As can be seen in Table 7, dose changes over a long period of treatment occurred more or less similarly with most medications in 2000. While benzodiazepines had the highest rates of both dose increase and dose reduction in 2000 and in 2010 (Table 8) the most dominant change in number of pills consumed during a long term treatment was seen with Z drugs.

## 4. Discussion

Our study reports on several major findings. First, of a huge, well managed database of over 4 million people insured (of whom 2.5 million and 3 million people in 2000 and 2010, resp., are adults), 8.7% in 2000 and 9.6% in 2010 consumed sleep medications. Of these, only 25% in 2000 and 21.4% in 2010 consumed them for longer than 6 months. Second, increasing age was associated with longer usage of hypnotics and a higher percent of sleep medication consumers were females. Minorities such as the Arab or Orthodox Jewish populations were significantly associated with lower and shorter consumption of hypnotics. Third, there is a strong trend to switch from monotherapy of benzodiazepines in 2000 to combined hypnotic medication treatment in 2010, along with reduction in benzodiazepine usage and increase in Z drugs usage. Finally, the rate of dose increase in long-term hypnotics treated (over 6 months) is relatively low (11.3% and 9.2% in 2000 and 2010, resp.), suggesting that tolerance is not common.

Insomnia is a very common disorder with many negative implications on health and quality of life. It negatively affects national economy and adds work load on the health system [2, 6–14]. Thus, it could be expected that many people will be treated. In our data, 8.7–9.6% of the adult CHS insured population is consuming hypnotics, of whom only about one quarter takes them on a regular long-term basis. This leads to the estimation that only about 2.5% of the population take hypnotics on a long-term basis. Given the high prevalence of insomnia [2, 5, 6], its burden on quality of life [5, 6, 9–14], and the relatively low accessibility and high cost of CBT [28], we expected that the number of chronic hypnotics users would be somewhat higher. Yet, our data indicate substantially higher number than those reported in the US. In a recent large, nationally representative study, it was reported that in the US there was an increasing prevalence of hypnotics use over time, from 2.0% in 1999-2000 to 3.5% in 2009-2010 [21]. Similar results were reported in other surveys as well [20, 29, 30], although some reports showed hypnotics usage of up to 16% of the population over 60 years of age [31]. It seems that the adult population in Israel consumes higher rates of hypnotics than in the US, despite the diseases' prevalence less than 15–30%. One explanation might be that compared to the US population, the Israeli population is more exposed to stressors as a result of the security issues and ongoing political conflicts in the Middle East. The reasons for relatively less than expected hypnotic usage are complex. We estimate that these relate partially to the concern regarding tolerance, side effects, and potential long term damage [18, 19, 32, 33]. Moreover, we believe that in the general population insomnia is still not considered a major problem or disease with substantial consequences. Many people still consider sleep as luxury or “nice to have” but not as a necessity. This in turn may lead to an under-diagnosis of insomnia [34, 35]. In addition, studies to prove that hypnotics induced sleep result in reduction of complications are very limited. Another option is that some of our patients have purchased hypnotics in the private market while given prescriptions by private physicians out of the CHS database. Since the cost of hypnotics is low this is an option, although not very high likely since the patients have trust in the CHS physicians and these visits are free of charge. We found that a higher percentage of hypnotics consumers were women, corroborating previous reports [2, 7, 21]. Similarly, our findings corroborate previous findings regarding age. The average age of hypnotics consumers was 70 years and was significantly lower in 2010 compared to 2000 (Table 1). We believe that the age lowering in hypnotics usage reflects two ongoing processes: (1) an increasing awareness of the importance of sleep and potential consequences of insomnia and (2) the development of new generation of hypnotics with less side effects and potential harm [36]. These two processes may lead to both increase in the usage of hypnotics and to using them in earlier ages.

The issue of hypnotics usage among minorities is interesting and was not deeply investigated. In 2000 and 2010 the percentage of hypnotics consumers among the Arab sector was relatively low (Table 2). We also found that chronic usage (6+ months) within the Arab sector was significantly lower than in other sectors (Table 4). Minorities in other studies were also reported to use less hypnotics than the average [29]. In addition, in many other diseases minorities in general, and the Arab or Orthodox Jewish population in Israel in particular, are late in diagnosis and treatment [37]. Thus, several explanations can be raised to understand this. Minorities and poverty can be associated with reduced health knowledge and/or accessibility, reduced understanding of the importance of sleep and insomnia, and lower ratio of physicians per population [38]. Since accessibility to health care is quiet good in Israel, and insomnia is covered under a general national coverage, we believe that there is less awareness to the importance of treating insomnia among Arabs and Orthodox Jews in Israel. Although there is a trend for improving awareness, there is still much to be done.

The duration of treatment is intriguing. Most people included in the research were treated for a period of 1–3 months (Table 3). Theoretically this might result from acute or short duration of insomnia, but realistically this is unlikely to be the reason. More likely, the reason is fear from addiction or tolerance to sleep medications. In most hypnotics leaflets (including zolpidem and zopiclone) it is stated that medications should be used on a short and not long-term basis. Furthermore, many physicians' message to the patients is that hypnotics carry a substantial risk of habituation and even addiction [8, 35, 36, 39]. Thus, patients themselves try to avoid long-term usage, and also pharmacists and physicians commonly discourage patients from chronic usage due to potential side effects [20, 32, 33, 40, 41]. Several studies have raised the concept of cancer or mortality associated with hypnotics usage [18, 19]. Thus, despite the chronic nature of insomnia most patients are treated only for limited periods of time. The chronic users are probably those with worse insomnia and increasing age, as indeed can be seen in our results (Table 3).

As our electronic data is well kept for more than 2 decades now, we could identify 10% presence of our hypnotics consumers (16,731 people), who were treated with hypnotics during both the years 2000 and 2010. Although we did not track their data in between, the fact that they used hypnotics through all 2000 and 2010 suggests they are chronic users and possibly used them continuously for over 10 years. With this reservation been said, if this is correct (presumably), it suggests that tolerance is less important than is thought to be. While in this 10-year period we had not retrieved data, we do have solid data for within-year periods of over 6 months (of both 2000 and 2010), showing no dose elevation and supporting the concept that tolerance to hypnotics is less common than thought to be. We found that, of all patients treated for longer than 6 months, only 11% in 2000 and 9% in 2010 required a dose increase. Although not a solid proof, based on a very large and reliable database, it suggests that most of our patients did not develop tolerance to the medication they took. This estimation is in concert with other several reports [16, 17, 42–44], although it definitely needs to be better tested. Several previous interventional studies that have proved how effective sleep medications are did not address treatment for periods longer than 6–12 months, even though for many patients insomnia can last for years [1, 7, 16].

We found that in 2010 only 54% of hypnotics consumers were treated with one type of medication compared to 78% in 2000 (Table 5). This means that there is a strong increase in prescription of combination of hypnotics from 2000 to 2010. This change in hypnotics consumption might be related to large variety of hypnotics that allow the medical community to prescribe more diverse and personally tailored medical treatments for insomnia. The Clinical Guideline for the Evaluation and Management of Chronic Insomnia in Adults published in JCSM, 2008 recommends treating insomnia with a combination of medications rather than escalating the dose of a single medication given [45]. It seems that the medical community in Israel follows these recommendations, which potentially may reduce tolerance. Similar results were recently reported in the NHANES study [21] that found that over 55% of sleep medication consumers reported also using other sedative medications.

We found that in both 2000 and 2010 most of the research population consumed benzodiazepines. In 2010 the percentage of consumption of Z drugs was higher than during 2000 (Table 5). These results are compatible with our expectations in light of Z drugs being developed during the last several years and the publication of studies starting from the year 2005, proving their efficacy and better safety profile [1, 3, 7, 8, 36]. Still, there might not be enough awareness among the older Israeli population, which continue to consume benzodiazepines for long periods without changing to newer Z drugs. For comparison, the NHANES study [21] found that in America Z drugs were the most commonly used category.

Z class medications are potentially less addicting. Based on recent clinical observations, addiction and tolerance during sleep medication treatment are generally rare [7]. This was reported predominantly with zolpidem [16, 17, 36]. Thus, the trend we observed of switching from benzodiazepines to more Z class medications is logical, although, in Israel unlike the US [21], it is a very slow process. Yet, in 2000 a smaller percent of Z drugs consumers needed to increase dose compared to benzodiazepines consumers. Surprisingly, in 2010 the opposite was found. This needs to be examined in future research. One potential explanation might be that the safer profile for Z drugs makes the patients and medical community more comfortable with increasing the dose in order to achieve better treatment results. Another potential explanation is that the policy in Israel is generally to start with low dose (i.e., 5 mg of zolpidem or 6.25 mg of Ambien CR) and if not effective enough increase the dose. Yet, most people that consumed hypnotics for longer than 6 months did not increase the dose. Demographic data, regarding hypnotics consumers who did increase the dose, did not show different gender, age, or sector distribution, and the current study cannot predict which patients are at higher risk of requesting increase of dosage. Of note, it was recently reported that a substantial portion of patients (up to 20%) increase their dosage, frequently without a physician recommendation, by adding a middle of the night pill ingestion [46]. In our study we tracked all medications dispensing but did not track the time of ingestion; thus we cannot state regarding a middle of the night hypnotic ingestion. It is plausible that those few patients who did increase dosage have added an additional dose in the middle of the night.

Our data has several strengths. First it consists of about 4 million people (2.5–3 million over 18 years of age) insured. This is one of the largest health care systems in the world. Second, the CHS database is very well managed, combining all patient related data regardless of physician or location of appointment or prescription. Third, Israel has a national coverage insurance; thus most hypnotics are covered equally to all the population. Therefore, we believe these findings are very reliable and representative despite the retrospective nature of our study. On the other hand, our study has several limitations. First, this was a retrospective study based on CHS database. As such, we could include only medications which are provided by CHS. Patients could potentially purchase different medication from outside the country or private pharmacies (if prescription is given by a private physician) paying with out-of-pocket money, in which cases they are not included in the database. Noncompliant patients or gaps between prescription and purchase were out of the scope of the current study, although this is a substantial problem in Israel especially among minorities [37, 47]. Second, we did not include medications such as OTC (antihistamines) and melatonin based medications (Circadin); thus our report may underestimate people using sleeping aids. Finally, the CHS population is slightly older with higher prevalence of chronic diseases than the general Israeli population; thus we cannot state completely accurately that this is representative of the entire population in Israel, although it represents the CHS insured population which is 55% of the population in Israel. Moreover, we did not look into the effects of comorbidities (medical or mental) or additional medication usage on hypnotics usage.

## 5. Conclusion

Despite the above mentioned limitations, we believe our data is very strong and based on over 4 million insured people in CHS in Israel. Based on this, 8.7–9.6% of the adult Israeli population consumes hypnotics, in 2000 and 2010, respectively. There is a strong increase in prescription of combination of medications from 2000 to 2010, with a tendency of increment in Z class medication and reduction in benzodiazepines. Hypnotics usage increases with age and in female gender, and most users do not increase dosage even after more than 6 months of daily usage, suggesting the absence of tolerance. The prevalence of hypnotics consumption within minorities is significantly lower than the general population.

## Figures and Tables

**Table 1:** Age average and median of sleep medication consumers.

Year	Age	
	Average	Median
2000	73.80^*∗*^	77.0^*∗*^
2010	67.26	70.0

^*∗*^ *p* < 0.0001 (2000 versus 2010).

**Table 2 tab2:** Percentage of sleep medication consumers in each population subgroup.

Year	Population subgroup		
	All others	Highly Orthodox Jews	Arabs
2000	10.3%^*∗*^	7.6%^*∗*^	3.0%
2010	11.3%^*∗*$^	7.5%^*∗*^	4.2%^$^

^*∗*^ *p* < 0.0001 (all others versus Orthodox Jews and Arabs and Orthodox Jew versus Arabs, in both 2000 and 2010); ^$^ *p* < 0.005 (within groups, change over time: 2000 versus 2010).

**Table 3 tab3:** Duration of consumption (in months) and age (years).

Number of months	Percentage (2000)	Percentage (2010)	Average age (2000)	Average age (2010)
1–3	63.1%	68.8%	70.56%	60.98^*∗*^
3–6	11.9%	9.8%	76.11^*∗*^	65.79^*∗*^
6+	25.0%	21.4%	79.35^*∗*^	68.80^*∗*^

^*∗*^ *p* < 0.005 (age between each two groups, for effects of duration of usage within each year and for effect of time within each duration group).

**Table 4 tab4:** Duration of consumption per sector.

	2000			2010		
	1–3 months	3–6 months	6+ months	1–3 months	3–6 months	6+ months
All others	61.9%	12.1%^*∗*^	26.0%^*∗*^	67.7%^*∗*^	10.1%^*∗*^	22.2%^*∗*^
Highly Orthodox Jews	61.4%	13.4%^*∗*^	25.3%^*∗*^	66.4%^*∗*^	11.2%^*∗*^	22.4%^*∗*^
Arabs	81.6%^$^	7.6%^*∗*$^	10.8%^*∗*$^	78.3%^*∗*$^	7.7%^*∗*$^	14.0%^*∗*$^

^*∗*^ *p* < 0.0001 (for all comparisons within sector subgroups); ^$^ *p* < 0.005 (within category based on duration of usage).

**Table 5 tab5:** Consumption distribution according to medication group type.

Year	Medication group type		
	Benzodiazepines	Antidepressant	Z drugs
2000	84.5%	13.4%	2.1%
2010	73.7%^*∗*^	16.5%^$^	9.8%^*∗*^

^*∗*^ *p* < 0.0001 (between 2000 and 2010), ^$^ *p* < 0.005 (2000 versus 2010).

**Table 6:** Change in the amount of sleep medications (dosage) consumption in patients consuming hypnotics for more than 6 months (of the entire study group).

Change	2000 [%]	2010 [%]
Decrease	7.0%	6.0%
No change	81.7%	84.9%
Increase	11.3%	9.2%

**Table 7:** Change tendency in the amount of sleep medications (dosage) consumption in patients consuming hypnotics for more than 6 months, according to medication group type (2000).

Medication group type	Change in dosage		
	Decrease	No change	Increase
Benzodiazepines	5.0%	85.7%	9.3%
Antidepressant	3.7%	88.7%	7.6%
Z drugs	2.4%	88.9%	8.7%

**Table 8:** Change in the amount of sleep medications (dosage) consumption in patients consuming hypnotics for more than 6 months, according to medication group type (2010).

Medication group type	Change in dosage		
	Decrease	No change	Increase
Benzodiazepines	5.6%	85.8%	8.6%
Antidepressant	7.8%	79.2%	13.1%
Z drugs	9.4%	77.0%	13.6%

## Additional Points

*Key Points. *While the prevalence of insomnia in the general population is estimated to be between 15% and 30%, about 9% of the population was found to consume hypnotics. There is a major increase in prescription of combination of medications between 2000 and 2010, with an increase in Z class medications use and reduction in benzodiazepines. Most patients treated for longer than 6 months did not escalate dosage, suggesting the absence of tolerance.

## References

[1] Silber M. H. (2005). Clinical practice. Chronic insomnia. *The New England Journal of Medicine*.

[2] Mai E., Buysse D. J. (2008). Insomnia: prevalence, impact, pathogenesis, differential diagnosis, and evaluation. *Sleep Medicine Clinics*.

[3] Neubauer D. N. (2007). The evolution and development of insomnia pharmacotherapies. *Journal of Clinical Sleep Medicine*.

[4] Dautovich N. D., McNamara J., Williams J. M., Cross N. J., McCrae C. S. (2010). Tackling sleeplessness: psychological treatment options for insomnia. *Nature and Science of Sleep*.

[5] Ohayon M. M. (2010). Nocturnal awakenings and difficulty resuming sleep: their burden in the European general population. *Journal of Psychosomatic Research*.

[6] Ohayon M. M., Sagales T. (2010). Prevalence of insomnia and sleep characteristics in the general population of Spain. *Sleep Medicine*.

[7] Wilson S. J., Nutt D. J., Alford C. (2010). British Association for Psychopharmacology consensus statement on evidence-based treatment of insomnia, parasomnias and circadian rhythm disorders. *Journal of Psychopharmacology*.

[8] Unbehaun T., Spiegelhalder K., Hirscher V., Riemann D. (2010). Management of insomnia: update and new approaches. *Nature and Science of Sleep*.

[9] Vgontzas A. N., Liao D., Bixler E. O., Chrousos G. P., Vela-Bueno A. (2009). Insomnia with objective short sleep duration is associated with a high risk for hypertension. *SLEEP*.

[10] Fernandez-Mendoza J., Vgontzas A. N., Liao D. (2012). Insomnia with objective short sleep duration and incident hypertension: the Penn State Cohort. *Hypertension*.

[11] Laugsand L. E., Vatten L. J., Platou C., Janszky I. (2011). Insomnia and the risk of acute myocardial infarction: a population study. *Circulation*.

[12] Kachi Y., Nakao M., Takeuchi T., Yano E. (2011). Association between insomnia symptoms and hemoglobin A1c level in Japanese men. *PLoS ONE*.

[13] Budhiraja R., Roth T., Hudgel D. W., Budhiraja P., Drake C. L. (2011). Prevalence and polysomnographic correlates of insomnia comorbid with medical disorders. *SLEEP*.

[14] Novak M., Mucsi I., Shapiro C. M., Rethelyi J., Kopp M. S. (2004). Increased utilization of health services by insomniacs—an epidemiological perspective. *Journal of Psychosomatic Research*.

[15] Garfinkel D., Zorin M., Wainstein J., Matas Z., Laudon M., Zisapel N. (2011). Efficacy and safety of prolonged-release melatonin in insomnia patients with diabetes: a randomized, double-blind, crossover study. *Diabetes, Metabolic Syndrome and Obesity: Targets and Therapy*.

[16] Krystal A. D., Erman M., Zammit G. K., Soubrane C., Roth T. (2008). Long-term efficacy and safety of zolpidem extended-release 12.5 mg, administered 3 to 7 nights per week for 24 weeks, in patients with chronic primary insomnia: a 6-month, randomized, double-blind, placebo-controlled, parallel-group, multicenter study. *SLEEP*.

[17] Kleykamp B. A., Griffiths R. R., McCann U. D., Smith M. T., Mintzer M. Z. (2012). Acute effects of zolpidem extended-release on cognitive performance and sleep in healthy males after repeated nightly use. *Experimental and Clinical Psychopharmacology*.

[18] Kripke D. F., Klauber M. R., Wingard D. L., Fell R. L., Assmus J. D., Garfinkel L. (1998). Mortality hazard associated with prescription hypnotics. *Biological Psychiatry*.

[19] Belleville G. (2010). Mortality hazard associated with anxiolytic and hypnotic drug use in the National Population Health Survey. *Canadian Journal of Psychiatry*.

[20] Vozoris N. T., Leung R. S. (2011). Sedative medication use: prevalence, risk factors, and associations with body mass index using population-level data. *Sleep*.

[21] Bertisch S. M., Herzig S. J., Winkelman J. W., Buettner C. (2014). National use of prescription medications for insomnia: NHANES 1999–2010. *SLEEP*.

[22] Tarasiuk A., Reznor G., Greenberg-Dotan S., Reuveni H. (2012). Financial incentive increases CPAP acceptance in patients from low socioeconomic background. *PLoS ONE*.

[23] Simon-Tuval T., Scharf S. M., Maimon N., Bernhard-Scharf B. J., Reuveni H., Tarasiuk A. (2011). Determinants of elevated healthcare utilization in patients with COPD. *Respiratory Research*.

[24] Simon-Tuval T., Reuveni H., Greenberg-Dotan S., Oksenberg A., Tal A., Tarasiuk A. (2009). Low socioeconomic status is a risk factor for CPAP acceptance among adult OSAS patients requiring treatment. *Sleep*.

[25] Safadi A., Etzioni T., Fliss D., Pillar G., Shapira C. (2014). The effect of the transition to home monitoring for the diagnosis of OSAS on test availability, waiting time, patients' satisfaction, and outcome in a large health provider system. *Sleep Disorders*.

[26] Saliba W., Rennert G. (2014). CHA2DS2-VASc score is directly associated with the risk of pulmonary embolism in patients with atrial fibrillation. *The American Journal of Medicine*.

[27] Hering E., Pritsker I., Gonchar L., Pillar G. (2009). Obesity in children is associated with increased health care use. *Clinical Pediatrics*.

[28] Cheung J. M. Y., Bartlett D. J., Armour C. L., Glozier N., Saini B. (2014). Insomnia patients' help-seeking experiences. *Behavioral Sleep Medicine*.

[29] Paulose-Ram R., Jonas B. S., Orwig D., Safran M. A. (2004). Prescription psychotropic medication use among the U.S. adult population: results from the third National Health and Nutrition Examination Survey, 1988–1994. *Journal of Clinical Epidemiology*.

[30] Calem M., Bisla J., Begum A. (2012). Increased prevalence of insomnia and changes in hypnotics use in England over 15 years: analysis of the 1993, 2000, and 2007 National Psychiatric Morbidity Surveys. *Sleep*.

[31] Neutel C. I., Patten S. B. (2009). Sleep medication use in Canadian seniors. *Canadian Journal of Clinical Pharmacology*.

[32] Chen P.-L., Lee W.-J., Sun W.-Z., Oyang Y.-J., Fuh J.-L. (2012). Risk of dementia in patients with insomnia and long-term use of hypnotics: a population-based retrospective cohort study. *PLoS ONE*.

[33] Kang D.-Y., Park S., Rhee C.-W. (2012). Zolpidem use and risk of fracture in elderly insomnia patients. *Journal of Preventive Medicine and Public Health*.

[34] Ram S., Seirawan H., Kumar S. K. S., Clark G. T. (2010). Prevalence and impact of sleep disorders and sleep habits in the United States. *Sleep and Breathing*.

[35] Bartlett D. J., Marshall N. S., Williams A., Grunstein R. R. (2008). Predictors of primary medical care consultation for sleep disorders. *Sleep Medicine*.

[36] Hoffmann F. (2013). Perceptions of German GPs on benefits and risks of benzodiazepines and Z-drugs. *Swiss Medical Weekly*.

[37] Rennert G., Peterburg Y. (2001). Prevalence of selected chronic diseases in Israel. *The Israel Medical Association Journal*.

[38] Robinson E., Mohilever J., Zidan J., Sapir D. (1986). Colorectal cancer: incidence, delay in diagnosis and stage of disease. *European Journal of Cancer and Clinical Oncology*.

[39] Voyer P., Préville M., Cohen D., Berbiche D., Béland S.-G. (2010). The prevalence of benzodiazepine dependence among community-dwelling older adult users in Quebec according to typical and atypical criteria. *Canadian Journal on Aging*.

[40] Ben-Hamou M., Marshall N. S., Grunstein R. R., Saini B., Fois R. A. (2011). Spontaneous adverse event reports associated with zolpidem in Australia 2001–2008. *Journal of Sleep Research*.

[41] Omvik S., Pallesen S., Bjorvatn B., Sivertsen B., Havik O. E., Nordhus I. H. (2010). Patient characteristics and predictors of sleep medication use. *International Clinical Psychopharmacology*.

[42] Dagan Y., Dushenat M., Silverman B. (2009). Chronic use of sleep medications—how serious is it in Israel?. *Harefuah*.

[43] Willems I. A. T., Gorgels W. J., Voshaar R. C., Mulder J., Lucassen P. L. (2013). Tolerance to benzodiazepines among long-term users in primary care. *Family Practice*.

[44] Allen R. P., Mendels J., Nevins D. B., Chernik D. A., Hoddes E. (1987). Efficacy without tolerance or rebound insomnia for midazolam and temazepam after use for one to three months. *The Journal of Clinical Pharmacology*.

[45] Schutte-Rodin S. L., Broch L., Buysee D., Dorsey C., Sateia M. (2008). Clinical guideline for the evaluation and management of chronic insomnia in adults. *Journal of Clinical Sleep Medicine*.

[46] Roth T., Berglund P., Shahly V., Shillington A. C., Stephenson J. J., Kessler R. C. (2013). Middle-of-the-night hypnotic use in a large national health plan. *Journal of Clinical Sleep Medicine*.

[47] Carel R. S., Brodsky I., Pillar G. (2015). Obstructive sleep apnea: comparison of syndrome severity and risk factors for adult Jewish and Arab males in Northern Israel. *Israel Medical Association Journal*.

